# Over one third of patients with symptomatic femoroacetabular impingement display femoral or acetabular version abnormalities

**DOI:** 10.1007/s00167-021-06643-3

**Published:** 2021-07-06

**Authors:** Zaki Arshad, Henry David Maughan, Karadi Hari Sunil Kumar, Matthew Pettit, Arvind Arora, Vikas Khanduja

**Affiliations:** 1 School of Clinical Medicine, University of Cambridge, Cambridge, UK; 2 Young Adult Hip Service, Addenbrooke’s, Cambridge University Hospital, Box 37, Hills Road, Cambridge, CB2 0QQ UK

**Keywords:** Femoroacetabular impingement, Femoral version, Acetabular version, Tibial torsion

## Abstract

**Purpose:**

The aim of this study was investigate the relationship between version and torsional abnormalities of the acetabulum, femur and tibia in patients with symptomatic FAI.

**Methods:**

A systematic review was performed according to PRISMA guidelines using the EMBASE, MEDLINE, PubMed and Cochrane databases. Original research articles evaluating the described version and torsional parameters in FAI were included. The MINORS criteria were used to appraise study quality and risk of bias. Mean version and torsion values were displayed using forest plots and the estimated proportion of hips displaying abnormalities in version/torsion were calculated.

**Results:**

A total of 1206 articles were identified from the initial search, with 43 articles, involving 8861 hips, meeting the inclusion criteria. All studies evaluating femoral or acetabular version in FAI reported ‘normal’ mean version values (10–25 °). However, distribution analysis revealed that an estimated 31% and 51% of patients with FAI displayed abnormal central acetabular and femoral version, respectively.

**Conclusion:**

Up to 51% of patients presenting with symptomatic FAI show an abnormal femoral version, whilst up to 31% demonstrate abnormal acetabular version. This high percentage of version abnormalities highlights the importance of evaluating these parameters routinely during assessment of patients with FAI, to guide clinical decision-making.

**Level of evidence:**

IV.

## Introduction

Femoroacetabular impingement (FAI) is characterised by an abnormal contact between the acetabulum and the femur, limiting range of motion and leading to hip pain and disability. Ganz et al. proposed that FAI may lead to the development of osteoarthritis of the hip joint [16]. FAI can be classified into three categories according to the specific pathomorphology involved. Cam type FAI represents asphericity of the femoral head due to abnormal morphology at the head neck junction [56]. Pincer-type FAI on the other hand, occurs due to over-coverage of the femoral head by the acetabulum and premature contact between the acetabulum and femoral neck [56]. Some patients may present with both of these abnormalities, known as mixed-typed FAI [56].

Increasingly, there is an interest in the role of acetabular and femoral version and tibial torsion in FAI. Ng et al. reported a significantly higher mean femoral version in those with symptomatic cam FAI as compared with healthy controls [38]. A study of 200 hips by Shin et al. demonstrated a significant correlation between combined index of acetabular and femoral version with both internal and external rotation of the hip [52]. Lerch et al. found that 68% of 538 hips presenting with FAI or dysplasia showed abnormal femoral and/or acetabular version [30]. A more recent study by Lerch et al. also found abnormal tibial torsion in 42% of patients with FAI and dysplasia [29].

Version abnormalities have gained interest because they may potentially influence the outcome following arthroscopic intervention for FAI [13]. Therefore, it is important to understand these abnormalities to ensure optimal decision-making. To the best of our knowledge, there are currently no systematic reviews characterising version and torsional deformities of the acetabulum, femur and tibia in FAI. The aim of this study, therefore, was to perform a systematic review investigating the relationship between version and torsional abnormalities of the acetabulum, femur and tibia in patients with primary FAI. Our hypothesis was that patients with symptomatic FAI displayed significant version abnormalities of the acetabulum or femur and tibial torsion.

## Materials and methods

### Search strategy

A systematic electronic search was performed by two reviewers independently using PubMed, OVID MEDLINE(R) (1946 to December 2020), Cochrane Library databases and OVID EMBASE (1974 to December 2020). Manual reference list checking of retrieved review articles was also conducted. Relevant terms such as ‘hip’, ‘pain’, ‘disorder’, ‘femoral version’, ‘femoral torsion’, ‘acetabular version’ and 'tibial torsion' were combined with Boolean operators (‘and’, ‘or’) to produce final searches. The systematic review title was registered in the Open Science Framework. All aspects of the Preferred Reporting Items for Systematic Reviews and Meta-Analysis (PRISMA) guidelines were followed whilst performing the systematic review [37]. The individual study inclusion and exclusion criteria were established a priori*.* Original research studies (observational, cohort, randomised control trials) evaluating femoral version, acetabular version or tibial torsion in Human patients with FAI were included. No specific control group was required for inclusion. Abstracts, review articles, commentaries and case reports were excluded. No language or date of publication criteria were imposed.

### Data management

Studies were imported into the Mendeley reference management software (© Mendeley Ltd, London, UK) to aid screening and selection.

### Selection process

Two reviewers independently (MZA and HDM) performed a two-stage title/abstract and full-text screening to identify eligible studies. Differences in opinion at any stage were resolved by discussion. A third, senior reviewer (VK) was consulted if there was a discrepancy and when no consensus was reached.

### Data extraction

An extraction spreadsheet containing the following headings was created in Microsoft Excel: (1) Author (2) Year of Publication (3) Title (4) Country of Origin (5) Aims (6) Participants including key demographics (7) Key findings (including mean acetabular and femoral version or tibial torsion). This spreadsheet was used by two authors (MZA and HDM) independently, to extract information from the first 10 studies. Discussion then took place to ascertain the suitability of the spreadsheet, following which it was decided to use the same spreadsheet to extract information from the remaining studies.

### Data synthesis

**Fig. 1:**
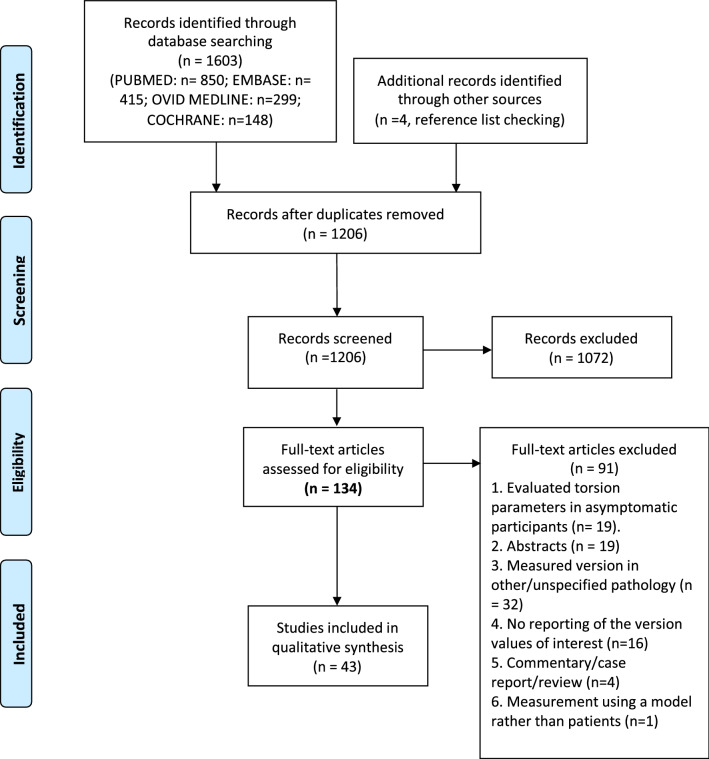
PRISMA flow diagram showing results of the search and screening process

Results of the search and screening processes are displayed in the PRISMA flow diagram (Fig. 1) [37]. Mean version values in the included studies, along with standard deviations are displayed in forest plots generated using R 4.0.0 software (R Foundation for Statistical Computing, Vienna, Austria). Acetabular version may be measured at a number of different axial planes along the cranio-caudal axis of the acetabulum [43]. Central acetabular version was defined as the angle between a line connecting the anterior and posterior acetabular rim at the level of the centre of the femoral head and a sagittal line [59]. Cranial acetabular version was defined as the measurement obtained when using any axial slice superior to the centre of the femoral head. Authors use a number of different positions when referring to cranial acetabular version and these are outlined (results, cranial version).

The normal range for femoral and central acetabular version of 10–25 ° originally proposed by Tönnis was used [59]. Missing standard deviation values were imputed using the guidelines outlined by Weir et al. and where study data was reported across two or more groups, these were combined following the methodology of Higgins and Green [22, 61]. The percentage of patients with version < 10 ° and > 25 ° was calculated. This is based on the assumption that version values are normally distributed, which has been demonstrated in previous studies [30, 31]. A qualitative synthesis of studies not included in meta-analysis or forest plots is provided.

### Risk of bias and quality of evidence

The Methodological Index for Non Randomized Studies (MINORS) criteria was used to assess the risk of bias and quality of all included studies [53]. The MINORS criteria consists of a 12 item checklist, each item given a score of 0 (not reported), 1 (inadequately reported) or 2 (adequately reported) [53]. The studies were scored against a maximum of 16 points for non-comparative studies and 24 points for comparative studies [53].

## Results

A total of 1206 studies were identified on the initial search and of these 43 articles involving 8861 hips finally met the inclusion criteria (Fig. 1). Results for the MINORS criteria are shown below (Table 1).

**Table 1:** Results of the MINORS criteria assessment

First author	Q1	Q2	Q3	Q4	Q5	Q6	Q7	Q8	Q9	Q10	Q11	Q12	N_H_	Total	Imaging modality
Audenaert [1]	2	2	2	2	0	2	2	2	2	2	2	2	30	22/24	CT
Bedi [2]	2	0	2	2	1	2	2	0	NA	NA	NA	NA	10	11/16	CT
Bedi [3]	2	0	2	2	0	2	2	0	NA	NA	NA	NA	8	10/16	CT
Bedi [4]	2	0	2	2	0	2	2	0	NA	NA	NA	NA	18	10/16	CT
Bouma [5]	2	0	2	2	0	2	2	0	2	2	2	2	55	18/24	CT
Cobb [8]	2	0	2	2	0	2	2	0	2	2	0	2	60	16/24	CT
Dandachli [9]	2	2	2	2	0	2	2	0	NA	NA	NA	NA	64	12/16	CT
De Pina Cabral [44]	2	0	2	2	0	2	2	0	NA	NA	NA	NA	35	10/16	CT
Ejnisman [11]	2	2	2	2	0	2	2	0	NA	NA	NA	NA	188	12/16	MRI
Fabricant [13]	2	2	2	2	0	2	2	0	NA	NA	NA	NA	243	12/16	CT
Ferro [14]	2	2	2	2	0	2	2	0	NA	NA	NA	NA	168	12/16	CT
Fritz [15]	2	2	2	2	0	2	2	0	2	2	2	2	380	20/24	MRI
Grammatopoulos [17]	2	0	2	2	0	2	2	2	2	0	2	2	49	16/24	CT
Hellman [19]	2	2	2	2	0	2	2	2	2	0	2	2	60	20/24	CT
Hetsroni [21]	2	2	2	2	0	2	2	0	NA	NA	NA	NA	197	12/16	CT
Jackson [23]	2	2	2	2	0	2	2	2	NA	NA	NA	NA	245	14/16	MRI
Kelly [24]	2	2	2	2	0	2	2	0	NA	NA	NA	NA	56	12/16	CT
Klingenstein [25]	2	2	2	2	0	2	2	0	2	2	2	2	646	20/24	CT
Lerch [30]	2	2	2	2	0	2	2	2	2	2	2	2	586	22/24	CT
Lerch [28]	2	2	2	2	0	2	2	0	2	2	0	2	84	18/24	CT
Lerch [29]	2	1	2	2	2	2	2	0	2	2	1	2	309	18/24	CT
Litrenta [31]	2	1	1	2	0	2	2	0	NA	NA	NA	NA	1449	10/16	MRI
Marostica [33]	2	1	2	2	0	2	2	0	NA	NA	NA	NA	51	11/16	MRI
Mascarenhas [34]	2	2	2	2	1	2	2	0	2	2	2	2	548	21/24	MRI
Masjedi [35]	2	0	0	2	0	2	2	0	2	0	0	2	71	12/24	CT
Milone [36]	2	0	2	2	0	2	2	0	NA	NA	NA	NA	100	10/16	CT
Ng [38]	2	0	2	2	2	2	2	0	2	2	1	2	43	19/24	CT
Ng [39]	2	0	2	2	2	2	2	0	2	2	2	2	20	22/26	CT
Ng [41]	2	0	2	2	2	2	2	0	2	2	0	2	54	18/24	CT
Ng [40]	2	0	2	2	0	2	2	0	2	2	0	2	57	16/24	CT
Ricciardi [45]	2	2	2	2	0	2	2	0	2	2	0	2	1776	18/24	CT
Ross [47]	2	2	2	2	0	2	2	0	NA	NA	NA	NA	50	12/16	CT
Ross [49]	2	2	2	2	0	2	2	0	NA	NA	NA	NA	50	12/16	CT
Ross [46]	2	2	2	2	2	2	2	0	2	2	2	2	102	22/24	CT
Ross [50]	2	2	2	2	0	2	2	0	NA	NA	NA	NA	50	12/16	CT
Ross [48]	2	2	2	2	0	2	2	0	NA	NA	NA	NA	17	12/16	CT
Schaefeller [51]	2	2	2	2	0	2	2	0	2	2	0	2	118	18/24	MRI
Shin [52]	2	2	2	2	2	2	2	0	NA	NA	NA	NA	200	14/16	CT
Sutter [54]	2	2	2	2	2	2	2	0	2	2	2	2	126	22/24	MRI
Tannast [55]	2	2	2	2	0	2	2	0	2	2	0	2	67	18/24	CT
Tibor [58]	2	2	1	2	0	2	2	0	NA	NA	NA	NA	112	11/16	MRI
Weinberg [60]	2	0	2	2	0	2	2	0	1	2	0	2	92	15/24	MRI (Patients), CT (Controls)
Yanke [62]	2	2	2	2	0	2	2	2	NA	NA	NA	NA	138	14/16	CT

Q1–Q12 refer to the question number on the MINORS checklist

*NA* not- applicable, *N*_*H*_ number of hips, *Imaging Modality* the imaging modality used to measure femoral version, acetabular version and tibial torsion specifically. *CT* computed tomography, *MRI* magnetic resonance imaging

### Femoral version

**Fig. 2:**
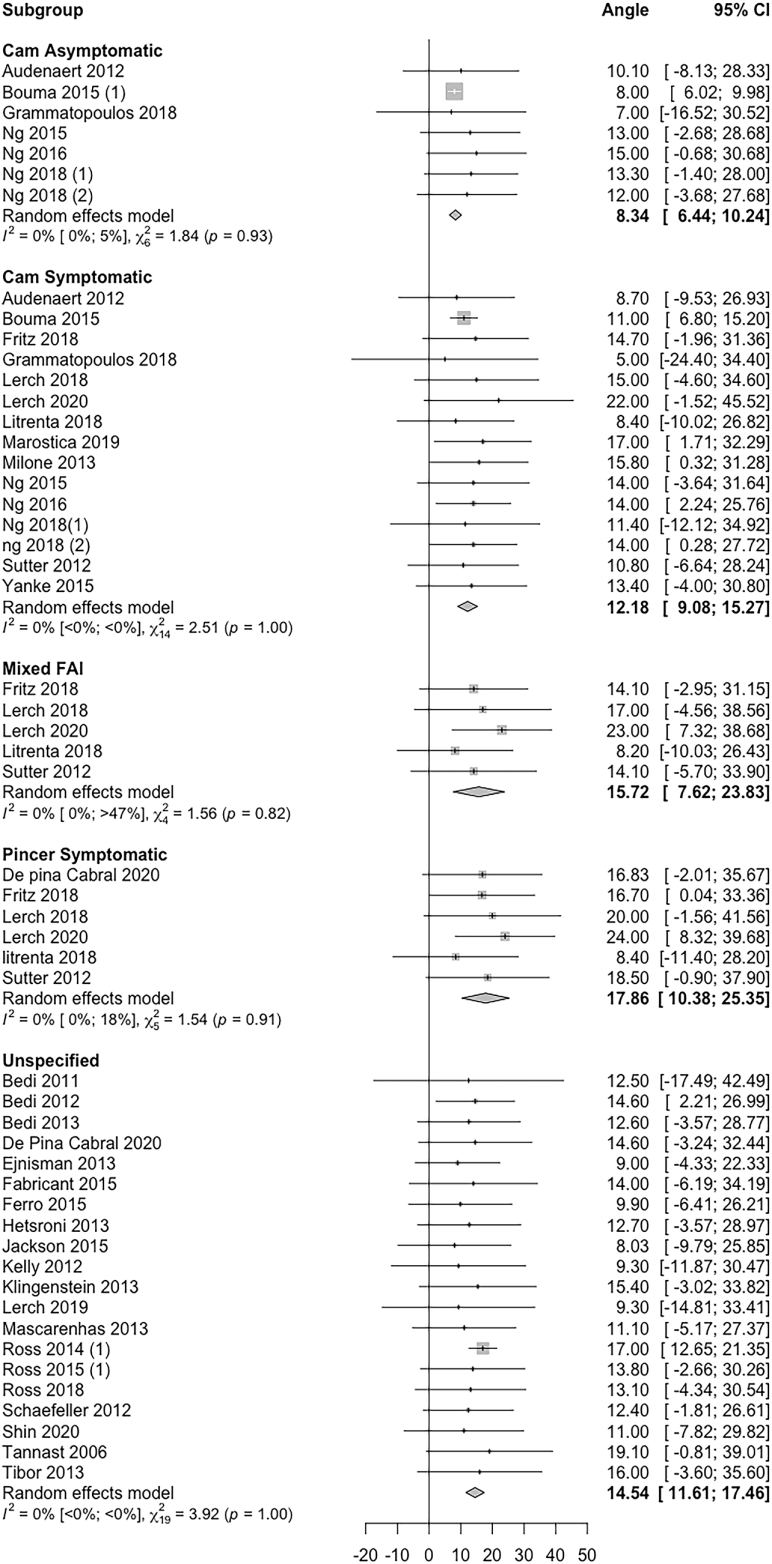
Forest plot showing an individual study level summary of mean and 95% confidence interval values for femoral version according to FAI sub-type. Sub-types used include cam asymptomatic, cam symptomatic, mixed, pincer symptomatic and unspecified (where the authors did not detail which specific FAI subtype was evaluated)

A total of 35 studies reported mean femoral version values in patients with various types of FAI. A forest plot displaying mean femoral version and 95% confidence intervals in these pathologies is displayed (Fig. 2). Majority (26/35, 74.2%) of the studies reported a mean femoral version value between 10 and 25 ^0^.

### Central acetabular version

**Fig. 3:**
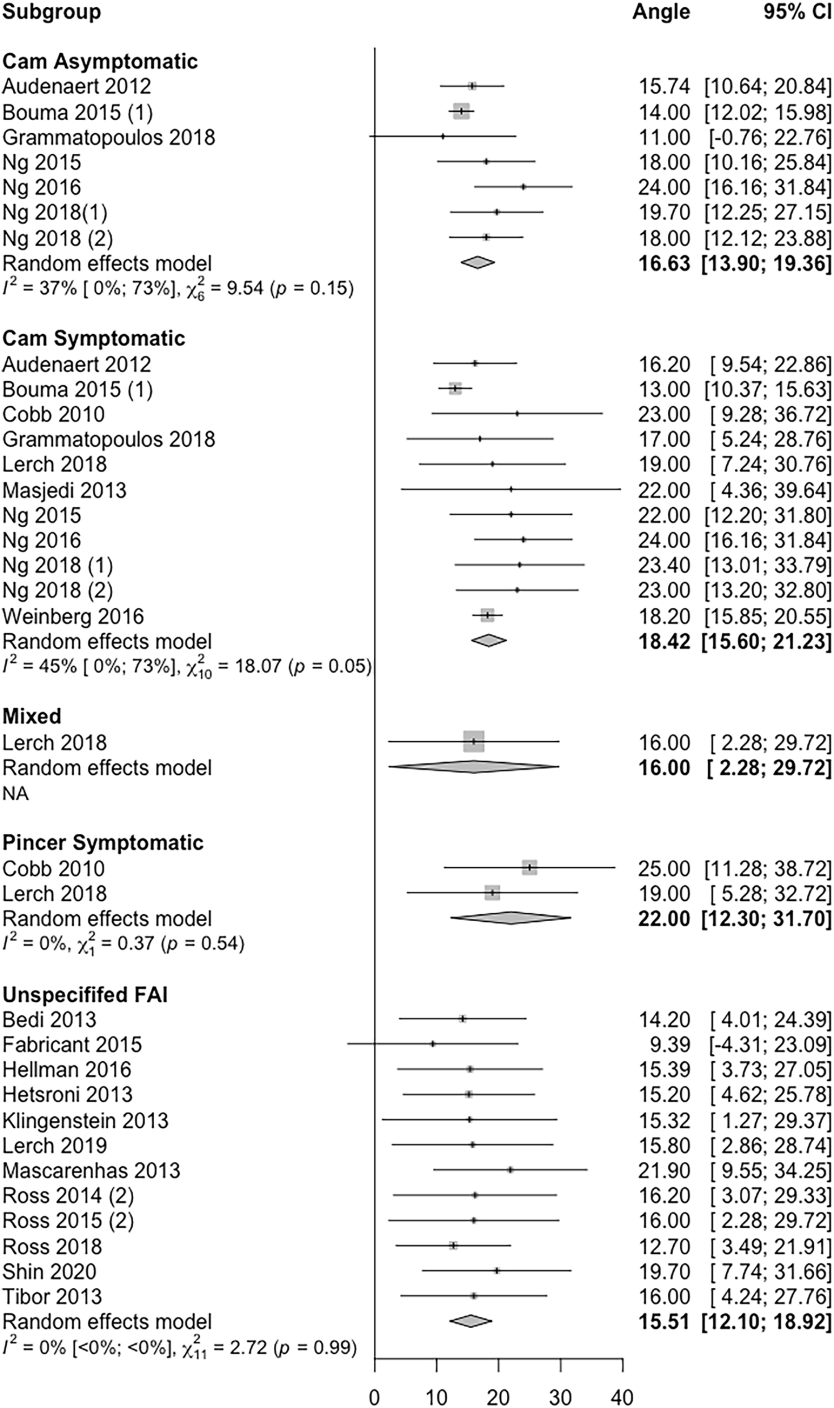
Forest plot showing an individual study level summary of mean and 95% confidence interval values for central acetabular version according to FAI sub-type. Sub-types used include cam asymptomatic, cam symptomatic, mixed, pincer symptomatic and unspecified (where the authors did not detail which specific FAI subtype was evaluated)

A total of 23 included studies reported mean central acetabular version values in different subtypes of FAI. A forest plot displaying mean central acetabular version and 95% confidence intervals in these pathologies is displayed in Fig. 3. All but one study (22/23, 95.7%) reported a mean acetabular version values in the normal range between 10 and 25 ^0^.

### Cranial acetabular version

**Fig. 4:**
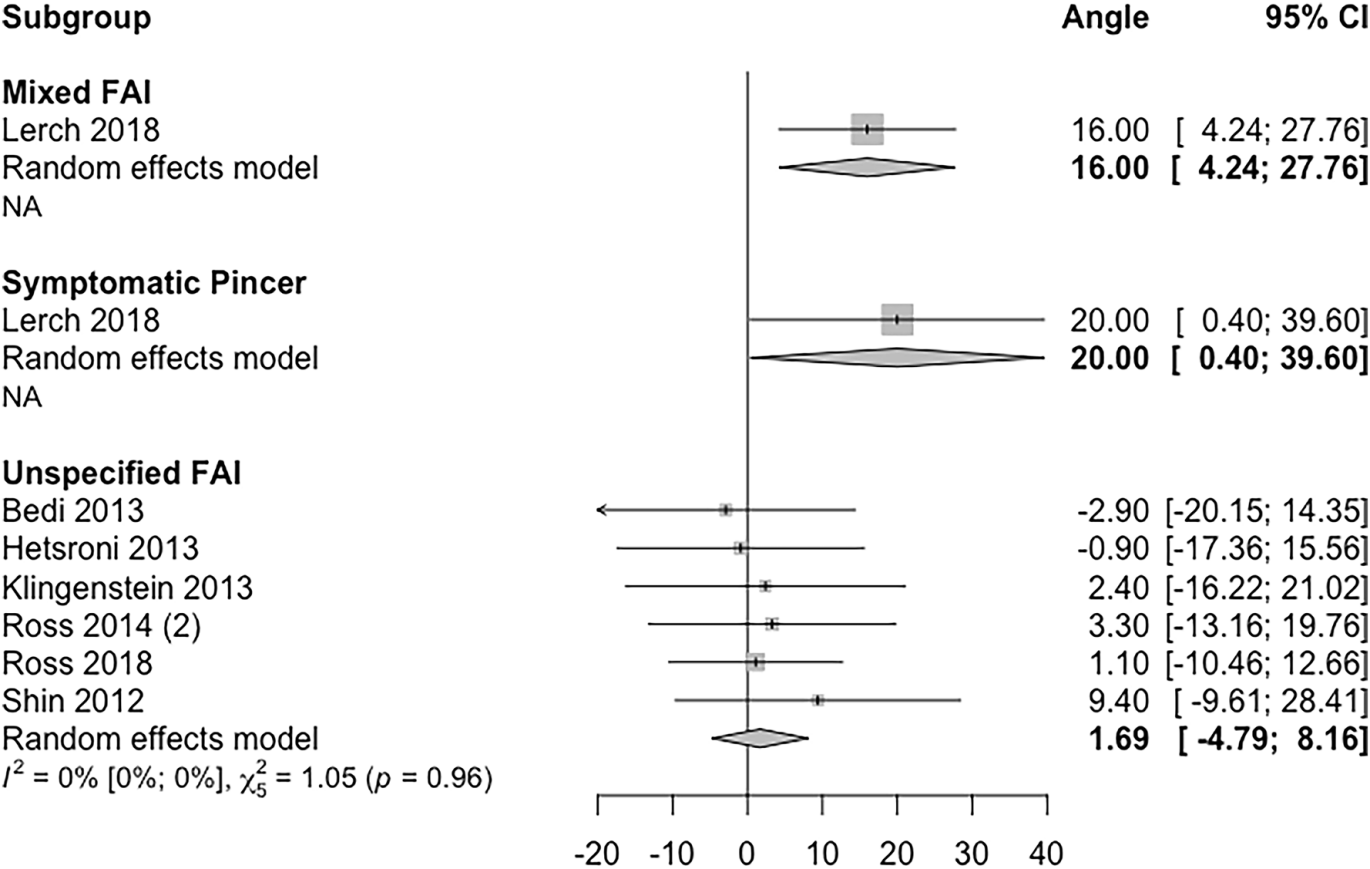
Forest plot showing an individual study level summary of mean and 95% confidence interval values for cranial acetabular version according to FAI sub-type. Sub-types used include mixed, pincer symptomatic and unspecified (where the authors did not detail which specific FAI subtype was evaluated)

Cranial acetabular version in FAI was reported by a total of seven studies (Fig. 4). Techniques used to measure cranial version varied in the included studies (using an axial slice 5 mm distal to acetabular roof, measurement at 1 o’clock position or 1:30 o’clock position) [4, 21, 25, 30, 48, 49, 52]. Due to this heterogeneity, no further distribution analysis was performed using these values. Figure 4 shows a forest plot displaying mean cranial acetabular version and 95% confidence intervals in various types of FAI. A total of six studies reported mean cranial acetabular version values less than 10 ° but did not specify the FAI subtype.

### Distribution analysis

Estimated distribution of femoral and acetabular version values in hips with FAI are shown (Tables 2 and 3).

**Table 2:** Table showing estimated distribution of femoral version values in hips with FAI

Pathology	Mean version (in degrees)	SD	Number of hips	FV < 10° (%)	FV > 25° (%)	Abnormal FV (%)
Symptomatic FAI	12.0	9.8	4660	41.9	9.2	51.2
Symptomatic Cam FAI	12.0	10.0	1224	42.4	9.4	51.8
Unspecified FAI	12.5	9.4	2514	39.8	9.2	49.0
Symptomatic Pincer	16.0	11.2	158	29.5	21.1	50.6
Mixed FAI	9.9	10.0	764	50.6	6.5	57.1

*FV* femoral version *SD* standard deviation

**Table 3:** Table showing estimated distribution of acetabular version values in hips with FAI

Pathology	Mean version (in degrees)	SD	Number of hips	AV < 10° (%)	AV > 25° (%)	Abnormal AV (%)
Symptomatic FAI	16.5	7.4	2269	18.9	12.6	31.4
Symptomatic Cam FAI	19.7	6.4	361	6.4	20.3	26.7
Unspecified FAI	15.7	7.4	1705	21.9	10.5	32.4
Symptomatic pincer	20.8	7.5	66	7.4	28.8	36.2

*AV* central acetabular version, *SD* standard deviation

### Studies not included in forest plots

Some studies were not included in forest plots due to paucity of data from similar studies and hence a summary of the findings is given Table 4.

**Table 4:** Table showing details of the studies not included in the forest plots

Author	Condition	Findings
Dandachali et al. [9]	Acetabular retroversion	Positive crossover sign in 41 of 64 (64.1%) hips
Klingenstein et al. [25]	Acetabular retroversion	Lower central acetabular version of 13.03° in patients with bilateral FAI compared to 15.86° in unilateral FAI
Ricciardi et al. [45]	Femoral version in extra-articular FAI	higher median femoral version of 21° in posterior, extra-articular FAI, compared to 8° in those with anterior extra-articular FAI
Lerch et al. [29]	tibial torsion in patients with symptomatic FAI	17% of patients had an increased tibial torsion of > 40° and 25% a decreased tibial torsion of < 25 Only 21% of hips in this study were found to show a combination of normal femoral version and normal tibial torsion

*AV* central acetabular version, *SD* standard deviation

## Discussion

51% of hips in patients with symptomatic FAI displayed abnormal femoral version, whilst 31% showed an abnormal acetabular version are the main findings of our study. These figures are similar to those demonstrated by Lerch et al., which found an abnormal femoral version in 52% of patients with FAI or dysplasia and abnormal acetabular version in 30% of patients [30]. The quality of studies included in this review is mixed, with the MINORS criteria revealing a number of studies show flaws in methodological rigour. Specifically, studies either fail to describe or do not use a consecutive enrolment design (question 5, Table 1). Furthermore, very few studies describe blinded evaluation of version parameters (question 8, Table 1). A significant proportion of included studies did not contain any control or comparison group. As such, it was not possible to evaluate questions 9–12 of the MINORS criteria.

Currently, there is no agreement on the ‘normal’ reference range values for femoral version, acetabular version and tibial torsion in patients with FAI. Thresholds for determining increased femoral version range from > 15 ^o^ to > 25 ° and decreased femoral version ranging from < 10 ° to < 0 ° [10, 12, 14, 26, 30, 59]. Similarly, authors have proposed various ranges for normal acetabular version including 10–25 ° and 13–20 ^o^ [30, 42]. Therefore, for the purpose of this review, the normal range for femoral and central acetabular version of 10–25 ° originally proposed by Tönnis was used [59].

Although the majority of included studies reported mean version values within ‘normal’ limits (Figs. 2 and 3), distribution analysis revealed that 31% of patients with FAI may have abnormal acetabular version < 10 ° or > 25 ° (Table 3) and up to 51% demonstrated abnormal femoral version < 10 ° or > 25 ^o^ (Table 2). More specifically, 42% had an excessive femoral retroversion < 10 °, whilst 19% had an abnormal acetabular version of < 10 °.

It remains to be seen whether these abnormalities represent a modifiable variable which may influence arthroscopic outcomes. Fabricant et al. found that although patients with femoral retroversion < 5 ° saw clinically important improvements in outcome following arthroscopic surgery, the outcomes were inferior to those with normal or increased femoral version [13]. On the other hand, Ferro et al., Lall et al., and Jackson et al., demonstrate no significant difference in outcomes in relation to femoral version [14, 23, 27]. Buller et al. reported that a complementary relationship existed between femoral and acetabular version whereby excessive acetabular retroversion may be compensated for by an increase in femoral anteversion, increasing impingement free range of motion [6]. Therefore, potentially in the above three studies, the influence of femoral version on outcomes may have been blunted by a compensatory acetabular version which was not specifically looked at. Furthermore, the study by Shin et al. found that the effect of a combined index of femoral and acetabular version was greater than that of femoral version alone [52]. Chaharbakshi et al. found patients with excessive femoral anteversion and borderline dysplasia showed inferior arthroscopic outcomes when compared to a matched control group [7].

Assessment of femoral version and tibial torsion is possible by clinical examination but not for acetabular version. It is therefore crucial that pre-operative imaging, such as a CT scan, is performed for patients with FAI, to gain a better understanding of the underlying deformities. Correction of any version abnormalities identified, through periacetabular or femoral osteotomy, together with cam or pincer excision, may potentially yield better outcomes in these patients. In addition, potentially some patients with significant version abnormality may require only correction of version abnormalities, without the need for arthroscopic intervention. Lerch et al. described abnormal femoral version (< 10 °, > 25 °) in over 74% of patients with clinical symptoms, but without any radiographic features of FAI [30]. Therefore, version abnormalities may indeed be the cause of hip pain in those presenting to the young adult hip clinic.

Studies have suggested specific differences in version may play a role in different subtypes of FAI [17, 30]. For example, a significantly lower femoral version of 15 ° has been demonstrated in patients with symptomatic Cam-FAI compared to asymptomatic controls (22°) [30]. Cam deformities have also been found in a considerable proportion of asymptomatic individuals [18, 57]. Furthermore, Gramamtopoulos et al. suggested that acetabular version may be one factor leading to the development of symptoms in patients with a cam deformity, showing a significantly higher acetabular version of 17 ° in symptomatic cam patients, compared to 11 ° in asymptomatic cam controls [17].

Our systematic review included studies which used both CT and MRI based measurement methods in the analysis. Although these two techniques have been found to show similar agreement compared to consecutive CT or MRI measurements, differences in version values gained using these techniques exist and there is a possibility that may be exacerbated when summarising studies [20]. Another factor to be noted is the failure of a number of studies to separate femoral/acetabular version measurements according to the FAI subtype. This meant we were unable to qualify the type of FAI where the version abnormality was noted.

It is important to note that even those studies identifying significant differences in version between patients with FAI and controls or between different types of FAI, reported mean version values in the patients within a normal range [17, 30, 38]. In such situation, there is a need to evaluate whether there are other potential causes for FAI such as extra-articular impingement or spinopelvic parameters [17, 34, 45]. Clinicians should take a more holistic approach and guide their treatment approach by considering how these measured parameters may collectively play a role in a patient’s symptoms. One way in which this has been done recently, is to combine femoral and acetabular version with the femoral neck-shaft angle, alpha angle and LCEA into a single value known as the ‘omega zone’. Bouma et al. found a significantly smaller omega zone in patients with cam-type FAI compared to controls [5]. We would, however, suggest that, although the use of such combined parameters can help quantify the rotational interaction between the femur and acetabulum, combining parameters may prevent clinicians from recognising and correcting important differences in individual parameters.

There is potentially a relationship between tibial torsion and femoral version; however, only one of the included studies reported tibial torsion values in patients with FAI [29]. Future research into FAI should evaluate tibial torsion in patients with FAI, to elucidate any potential role that this parameter may play in the management of FAI.

This systematic review was conducted in the rigorous manner outlined by PRISMA. However certain limitations must also be acknowledged. First, when estimating the percentage of patients showing abnormalities in femoral and acetabular version, every cohort was assumed to show a normal distribution of these values. Although large studies have previously shown a normal distribution, this may not be true for all study cohorts, particularly those of smaller size [30, 31]. Furthermore, different measurement methods, including CT and MRI were used in included studies. Although studies have shown a high degree of consistency between these measurement methods, differences may still exist [20, 32]. Unfortunately, it was not possible to perform a meta-analysis investigating differences in femoral/acetabular version between FAI subtypes due to the inherently large degree of heterogeneity.

## Conclusion

Up to 51% of patients presenting with symptomatic FAI show an abnormal femoral version, whilst up to 31% demonstrate abnormal acetabular version. These abnormalities may represent a modifiable variable with an influence on arthroscopic outcomes. As such, consideration of these morphological parameters in the assessment and management of patients with FAI is a crucial step in the development of a holistic arthroscopic approach, taking into account both pathomorphology and patho-alignment.
